# Sensitivity and specificity of the antigen-based anterior nasal self-testing programme for detecting SARS-CoV-2 infection in schools, Austria, March 2021

**DOI:** 10.2807/1560-7917.ES.2021.26.34.2100797

**Published:** 2021-08-26

**Authors:** Peter Willeit, Benoît Bernar, Christoph Zurl, Mariam Al-Rawi, Andrea Berghold, David Bernhard, Wegene Borena, Christian Doppler, Reinhold Kerbl, Alwin Köhler, Robert Krause, Bernd Lamprecht, Johannes Pröll, Hannes Schmidt, Ivo Steinmetz, Evelyn Stelzl, Heribert Stoiber, Dorothee von Laer, Johannes Zuber, Thomas Müller, Volker Strenger, Michael Wagner

**Affiliations:** 1 Clinical Epidemiology Team, Department of Neurology, Medical University of Innsbruck, Innsbruck, Austria; 2 Department of Public Health and Primary Care, University of Cambridge, Cambridge, United Kingdom; 3 Department of Pediatrics I, Medical University of Innsbruck, Innsbruck, Austria; 4 Section of Infectious Diseases and Tropical Medicine, Department of Internal Medicine, Medical University of Graz, Graz, Austria; 5 BioTechMed-Graz, Graz, Austria; 6 Department of Paediatrics and Adolescent Medicine, Division of General Paediatrics, Medical University of Graz, Graz, Austria; 7 Vienna Covid-19 Detection Initiative, Vienna, Austria; 8 Max Perutz Labs, University of Vienna, Vienna, Austria; 9 Institute for Medical Informatics, Statistics and Documentation, Medical University of Graz, Graz, Austria; 10 Center for Medical Research, Faculty of Medicine, Johannes Kepler University Linz, Linz, Austria; 11 Division of Pathophysiology, Institute of Physiology and Pathophysiology, Johannes Kepler University Linz, Linz, Austria; 12 Institute of Virology, Medical University Innsbruck, Innsbruck, Austria; 13 Department of Paediatrics and Adolescent Medicine, LKH Hochsteiermark, Leoben, Austria; 14 Department of Pulmonology, Kepler-University-Hospital, Faculty of Medicine, Johannes Kepler University Linz, Linz, Austria; 15 Centre for Microbiology and Environmental Systems Science, Department of Microbiology and Ecosystem Science, University of Vienna, Vienna, Austria; 16 Diagnostic and Research Institute of Hygiene, Microbiology and Environmental Medicine, Medical University of Graz, Graz, Austria; 17 IMP - Research Institute of Molecular Pathology, Vienna, Austria; 18 Department of Paediatrics and Adolescent Medicine, Division of Pediatric Pulmonology and Allergology, Medical University Graz, Graz, Austria; 19 Center for Microbial Communities, Department of Chemistry and Bioscience, Aalborg University, Aalborg, Denmark

**Keywords:** SARS-CoV-2, Antigen-based test, PCR-based test, School, Sensitivity, Specificity, Children

## Abstract

This study evaluates the performance of the antigen-based anterior nasal screening programme implemented in all Austrian schools to detect SARS-CoV-2 infections. We combined nationwide antigen-based screening data obtained in March 2021 from 5,370 schools (Grade 1–8) with an RT-qPCR-based prospective cohort study comprising a representative sample of 244 schools. Considering a range of assumptions, only a subset of infected individuals are detected with the programme (low to moderate sensitivity) and non-infected individuals mainly tested negative (very high specificity).

Early recognition and isolation of infected individuals are crucial in containing the coronavirus disease (COVID-19) pandemic. Rapid antigen tests deliver timely results, can be performed without laboratory resources, and are widely used in screening programmes for severe acute respiratory syndrome coronavirus 2 (SARS-CoV-2) [1-3]. As transmission and outbreaks occur in educational settings [4], a nationwide screening programme was introduced at Austrian schools in January 2021 that implemented regular antigen-based anterior nasal self-tests for pupils, teachers and administrative staff [5].

The present study aimed to evaluate the performance of the antigen-based screening programme for detecting SARS-CoV-2 infections in Austrian schools. The design of the study is outlined in Figure 1. To provide context, the mean 7-day community incidence during the study period was 198 per 100,000 individuals across all ages.

**Figure 1:**
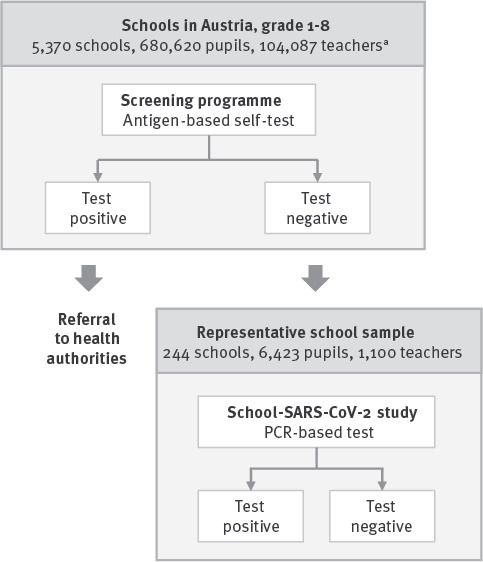
Study design for evaluating the performance of the rapid antigen test-based SARS-CoV-2 screening programme in schools, Austria, March 2021

^a^ Included administrative staff.

## Nationwide rapid antigen test-based screening programme

The first component used aggregate data from the nationwide rapid antigen test-based screening programme (Figure 1) provided by the Federal Ministry of Education, Science and Research. Data were collected in Weeks 9–11 at primary schools (Grades 1–4) and lower secondary schools (Grades 5–8) and involved 680,620 pupils and 104,087 teachers and administrative staff from 5,370 schools. Participants were typically asymptomatic or pauci-symptomatic since pupils and teachers with symptoms were strictly advised to stay at home. Participation in the screening programme was free of charge, obligatory for in-person teaching, and required written informed consent. Participants conducted self-testing under the supervision of a teacher, twice per week in primary schools (Mondays, Wednesdays) and once per week in lower secondary schools (Mondays or Wednesdays as classes were split in two fixed cohorts and taught in school in a staggered manner). Mean participation rate over Weeks 9–11 was 98.5% (range: 96.4–98.8). 

The screening programme used two rapid antigen tests licenced for anterior nasal self-testing (Lepu Medical SARS-CoV-2 Antigen Rapid Test, Lepu Medical Technology, Beijing, China [6]; and Flowflex SARS-CoV-2 Antigen Rapid Test, ACON Biotech, Hangzhou, China [7]). The mean percentage tested positive each week is provided in the Table. Positively tested individuals were sent home immediately and referred to the local health authorities as suspected cases for further testing.

**Table:** Number of participants and percentage^a^ tested positive, SARS-CoV-2 screening studies in schools, Austria, March 2021 (n = 784,707)

	Antigen-based screening programme		School-SARS-CoV-2 study		
	Participants (n)	% positive^a^	Participants (n)	% positive^b^	95% CI
Pupils in primary schools	360,948	0.106	4,161	0.26	0.13–0.52
Pupils in lower secondary schools	319,672	0.063	2,262	0.13	0.04–0.41
Teachers at primary/lower secondary schools	104,087^c^	0.292	1,100	0.18	0.05–0.73

CI: confidence interval.

^a^ For the antigen-based screening programme, the percentage tested positive was the mean percentage of participants tested positive each week weighted to the number of participating individuals. The percentages positive are provided without 95% CI because all pupils and educational staff were involved in the programme, rather than drawing a representative sample. The percentages of participants tested positive in Weeks 9, 10 and 11 were 0.088, 0.090 and 0.140 among pupils in primary schools, 0.055, 0.053 and 0.079 among pupils in lower secondary schools and 0.309, 0.313 and 0.254 among teachers and administrative staff in primary/lower secondary schools.

^b^ 95% CI of prevalence estimates in the Austrian School-SARS-CoV-2 study were calculated from robust standard errors estimated based on clustered Sandwich estimators.

^c^ Included administrative staff.

All participants in the School-SARS-CoV-2 study had before the study tested negative in the antigen-based screening programme.

## Austrian School-SARS-CoV-2 study

The second component of this study used data from the Austrian School-SARS-CoV-2 study (Figure 1), a prospective cohort study involving pupils and teachers from a representative sample of Austrian schools (Grade 1–8). Every 3–5 weeks during periods of the school year 2020/21 not affected by school closures, participants were tested for SARS-CoV-2 infection using gargling, pooling and in-house RT-qPCR analysing multiple target genes (i.e. ORF1b, ORF10 and N, the latter being detected by two different assays N1 and N2). For all positive samples, at least two viral genes were detected. In addition, an RT-qPCR test for human RNA was included in all assays. The RT-qPCR controls included RNA extracts (i) from a previously tested positive sample with a target Cq value of 25 or synthetic SARS-CoV-2 RNA control (1 × 10^4^ molecules per reaction; Twist Biosciences, San Francisco, United States), (ii) from a previously tested negative sample and (iii) a no-template control. Further details on study design and methodology have been published previously [8]. 

**Figure 2:**
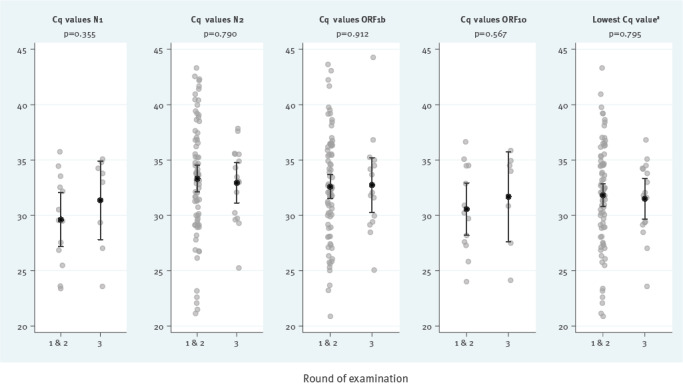
Cq values of unpooled RT-qPCR of positively tested participants in Round 3 compared with Rounds 1 and 2, School-SARS-CoV-2 study, Austria, March 2021 (n = 92 in Rounds 1 and 2, n = 16 in Round 3)

^a^ Lowest value among the different Cq values of the RT-qPCR target genes.

Our analysis focused on Round 3 of the Austrian School-SARS-CoV-2 study, which was conducted between 1 and 18 March 2021 (Weeks 9–11) and included 6,423 pupils and 1,100 teachers (from 244 schools) who had previously tested negative in the rapid antigen test-based screening programme. Key findings on the prevalence of RT-qPCR-detected SARS-CoV-2 infection are shown in the Table. Overall prevalence did not differ significantly by time since the last antigen-based self-test: Prevalence was 0.27% (8/2,985) in children and teachers with a negative self-test on the same day vs 0.18% (8/4,538) for people with earlier rapid antigen testing (85% tested on the preceding day), corresponding to an odds ratio of 1.51 (95% confidence interval: 0.53–4.37; p = 0.435). There was no significant difference in Cq values among positively tested individuals in Round 3 compared with earlier rounds when no antigen-based screening programme had been in place (Figure 2). Of note, eight of 14 pupils and both teachers who tested positive by RT-qPCR had Cq values > 30 for all tested targets.

Grey circles: Cq values of the individual RT-qPCR positive cases; black dots and vertical lines: their arithmetic means and 95% confidence intervals. N, ORF1b, and ORF10 are RT-qPCR target genes; N1 and N2 represent two different RT-qPCR assays for detection of the N-gene. A two-sample t-test was used to test for difference in Cq values between positively tested participants at Round 1 and 2 compared with positively tested participants at Round 3. Round 1 took place from 28 September to 22 October 2020 (40 RT-qPCR positive cases), Round 2 from 10 to 16 November 2020 (52 positive cases) and Round 3 from 1 to 18 March 2021 (16 positive cases).

## Estimated sensitivity and specificity of the Austrian antigen-based screening programme

**Figure 3:**
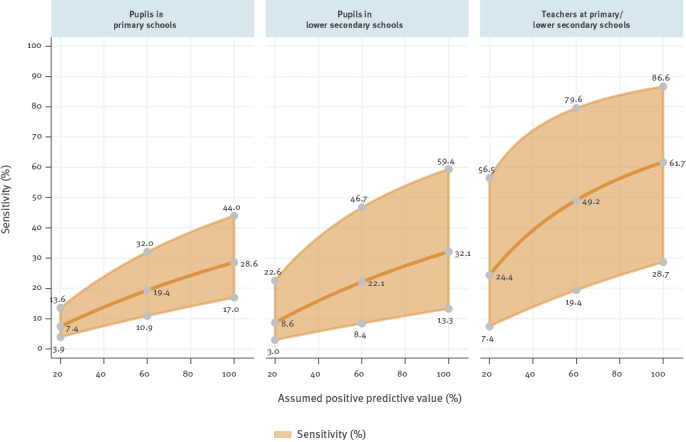
Modelled sensitivity of antigen-based anterior nasal self-testing programme assuming a range of positive predictive values and point estimates of the negative predictive value detected in the School-SARS-CoV-2 study, Austria, March 2021

We modelled sensitivity and specificity of the antigen-based screening programme at Austrian schools by (i) combining findings from the two data sources, (ii) assuming a range of positive predictive values for the rapid antigen test (20–100%) and (iii) using point estimates and 95% confidence interval limits in the Austrian School-SARS-CoV-2 study as estimates of the negative predictive value (Figure 3). For instance, assuming a positive predictive value of 20%, sensitivity is expected in the range of 3.9–13.6% among pupils in primary schools, 3.0–22.6% among pupils in lower secondary schools and 7.4–56.5% among teachers. Corresponding sensitivity ranges are 10.9–32.0%, 8.4–46.7% and 19.4–79.6% assuming a positive predictive value of 60%, and 17.0–44.0%, 13.3–59.4% and 28.7–86.6% assuming a perfect positive predictive value of 100%. The model indicated high specificity values at 20%, 60% and 100% assumed positive predictive values of 99.92%, 99.6% and 100% for pupils in primary schools, 99.95%, 99.7% and 100% for pupils in lower secondary schools and 99.77%, 99.88% and 100% for teachers at primary/secondary schools.

Dark orange: point estimates; light orange areas: 95% confidence interval limits. The numbers next to the grey circles are exemplary sensitivity values at positive predictive values of 20%, 60% and 100%.

## Ethical statement

Ethical approval was not required for the data from the nationwide rapid antigen test-based screening programme because we used aggregate data only. The Austrian School-SARS-CoV-2 study received ethics approvals by the ethics committees of the Medical University of Graz (no. 32–672 ex 19/20), Medical University of Innsbruck (no. 1319/2020), the Johannes Kepler University of Linz (no. 1222/2020), and the University of Vienna (no. 00591/2020). Participants or their legal representative provided written informed consent.

## Discussion

The present study scrutinised the antigen-based screening programme introduced at Austrian schools in early 2021 to detect SARS-CoV-2 cases. While self-collected anterior nasal swabs can be easily performed, were well accepted by the children in our study and help increase testing capacities, knowledge about their performance in real-world settings are required to guide further decision-making.

While several studies exist on sensitivity and specificity of rapid antigen tests in adults [2], a recent Cochrane review identified a lack of large-scale data on the performance of serial antigen-based screening strategies in asymptomatic school-aged children [3]. Across various modelled scenarios, we observed lower sensitivity but similar specificity in children compared with prior studies in adults [3]. Collection of specimens is not performed by trained medical staff but by pupils in Grades 1–8 and their teachers, which might substantially impact the detection rate. Furthermore, sensitivity of antigen-based tests is highest in symptomatic patients, in whom viral loads are higher [9,10]. Less is known about viral loads in the anterior nasal region compared with the throat in asymptomatic people and whether age-specific differences exist, but some data indicate that virus presence in the anterior nose can be delayed in asymptomatic people, leading to a lower detection rate in the early phase of infection [11,12]. Consequently, an a priori reduced sensitivity can be assumed in our cohort of asymptomatic individuals. Indeed, many individuals who falsely tested negative in the antigen-based screening programme had Cq values > 30 at the moment of testing. While this may indicate a lower viral load and infectivity at this stage, Cq trajectories in positive cases over time are unclear, and some cases in the early phase of infection might have subsequently developed lower Cq values. 

Our study has strengths and limitations. By combining nationwide data on the antigen-based screening programme with RT-qPCR testing in a carefully chosen representative sample of schools, we provide a unique insight into the potential and limitations of one of the key measures to mitigate SARS-CoV-2 transmission in schools. However, our findings may not be generalisable to other countries and antigen tests. Also, for reasons of data protection, only aggregate data were available from the screening programme, precluding more detailed analyses. Furthermore, health authorities could not provide data on how frequently a positive rapid antigen test was confirmed with RT-qPCR. To account for this limitation, our modelling involved a range of positive predictive values.

## Conclusion

Our study indicates that only a subset of infected individuals are detected with the antigen-based screening programme at Austrian schools (low to moderate sensitivity). Non-infected individuals were largely tested negative (very high specificity). Given the low-to-moderate sensitivity of antigen-based anterior nasal self-testing particularly in children, additional measures such as face masks or ventilation are important to prevent secondary cases, especially in periods with high incidence. Furthermore, as a mitigation measure, switching SARS-CoV-2 screening in schools to RT-qPCR based approaches should be preferred, where logistically feasible.
